# THE IMPACT OF AGE-SPECIFIC PSYCHOLOGICAL SELF-SUFFICIENCY ON ECONOMIC WELL-BEING AMONG OLDER WORKER JOB SEEKERS

**DOI:** 10.1093/geroni/igae098.3242

**Published:** 2024-12-31

**Authors:** Mina Lee, Hyesu Yeo, Philip Hong

**Affiliations:** Binghamton University, Binghamton, New York, United States; University of Georgia, Athens, Georgia, United States; University of Georgia, Athens, Georgia, United States

## Abstract

**Aim:**

Federal and state workforce policies prioritize Economic Self-Sufficiency (ESS) but often overlook crucial psychological dimensions. Psychological Self-Sufficiency (PSS) provides an empowerment framework, especially for low-income jobseekers, integrating psychological and economic well-being. While PSS represents a transformative path from Employment Barrier (EB) to Employment Hope (EH) and positively impacts ESS, the age-specific impact of PSS measures on economic well-being remains unexplored. This study validates PSS measures and investigates their relationship with ESS among older worker job seekers.

**Methods:**

Data were collected from 1006 participants aged 50+, engaged with community-based agencies offering employment training services (2012-2016. Exploratory Factor Analysis (EFA) and Confirmatory Factor Analysis (CFA) identified older-worker PSS using 27 EB and 24 EH items. ESS was assessed using the 15-item WEN ESS scale. Structural Equation Modeling (SEM) examined the relationship between older-worker PSS and ESS pre- and post-program participation.

**Results:**

EFA and CFA revealed a 16-item 4-factor EB and a 15-item 3-factor EH structure. EB factors included human capital, personal balance, physical and mental health, and labor market exclusion barriers, while EH factors encompassed self-assurance, skill and resource utilization, and goal orientation. SEM indicated significant PSS, highlighting the transformative path from EB to EH, significantly affecting ESS with EH fully mediating EB.

**Conclusion:**

This study highlights the distinct age-specific composition of PSS factors among low-income older workers, emphasizing the need for age-friendly intervention strategies by workforce development practitioners. By enhancing psychological self-sufficiency, these interventions can contribute to the economic well-being of older workers in the context of employment transition.

